# Molecular mechanism for endo-type action of glycoside hydrolase family 55 endo-β-1,3-glucanase on β1-3/1-6-glucan

**DOI:** 10.1016/j.jbc.2023.105294

**Published:** 2023-09-27

**Authors:** Tomoya Ota, Wataru Saburi, Takayoshi Tagami, Jian Yu, Shiro Komba, Linda Elizabeth Jewell, Tom Hsiang, Ryozo Imai, Min Yao, Haruhide Mori

**Affiliations:** 1 Research Faculty of Agriculture, Hokkaido University, Sapporo, Japan; 2 Graduate School of Life Science, Hokkaido University, Sapporo, Japan; 3 Institute of Food Research, National Agriculture and Food Research Organization, Tsukuba, Japan; 4 St. John's Research and Development Center, Agriculture and Agri-Food Canada, St John's, Newfoundland and Labrador, Canada; 5 School of Environmental Sciences, University of Guelph, Guelph, Canada; 6 Institute of Agrobiological Sciences, National Agriculture and Food Research Organization, Tsukuba, Japan

**Keywords:** glycoside hydrolase family 55, laminarin, endo-β-1,3-glucanase, enzyme structure, molecular docking, glycoside hydrolase, enzyme mechanism, polysaccharide

## Abstract

The glycoside hydrolase family 55 (GH55) includes inverting exo-β-1,3-glucosidases and endo-β-1,3-glucanases, acting on laminarin, which is a β1-3/1-6-glucan consisting of a β1-3/1-6-linked main chain and β1-6-linked branches. Despite their different modes of action toward laminarin, endo-β-1,3-glucanases share with exo-β-1,3-glucosidases conserved residues that form the dead-end structure of subsite −1. Here, we investigated the mechanism of endo-type action on laminarin by GH55 endo-β-1,3-glucanase MnLam55A, identified from *Microdochium nivale*. MnLam55A, like other endo-β-1,3-glucanases, degraded internal β-d-glucosidic linkages of laminarin, producing more reducing sugars than the sum of d-glucose and gentiooligosaccharides detected. β1-3-Glucans lacking β1-6-linkages in the main chain were not hydrolyzed. NMR analysis of the initial degradation of laminarin revealed that MnLam55A preferentially cleaved the nonreducing terminal β1-3-linkage of the laminarioligosaccharide moiety at the reducing end side of the main chain β1-6-linkage. MnLam55A liberates d-glucose from laminaritriose and longer laminarioligosaccharides, but *k*_cat_/*K*_m_ values to laminarioligosaccharides (*≤*4.21 s^−1^ mM^−1^) were much lower than to laminarin (5920 s^−1^ mM^−1^). These results indicate that β-glucan binding to the minus subsites of MnLam55A, including exclusive binding of the gentiobiosyl moiety to subsites −1 and −2, is required for high hydrolytic activity. A crystal structure of MnLam55A, determined at 2.4 Å resolution, showed that MnLam55A adopts an overall structure and catalytic site similar to those of exo-β-1,3-glucosidases. However, MnLam55A possesses an extended substrate-binding cleft that is expected to form the minus subsites. Sequence comparison suggested that other endo-type enzymes share the extended cleft. The specific hydrolysis of internal linkages in laminarin is presumably common to GH55 endo-β-1,3-glucanases.

β-1,3-Glucanases (EC 3.2.1.6, 3.2.1.39, or 3.2.1.58) are found in bacteria, fungi, plants, and marine organisms. These enzymes hydrolyze various β1-3-glucans such as linear β1-3-glucans, curdlan and paramylon (1); β1-3/1-6-glucans, laminarin (2) (Fig. 1) and scleroglucan (3); and β1-3/1-4-glucan (4) to release d-glucose and/or oligosaccharides. In the carbohydrate-active enzymes database (5), β-1,3-glucanases belong to glycoside hydrolase family 3 (GH3), GH5, GH8, GH9, GH16, GH17, GH55, GH64, GH81, GH128, GH132, GH152, GH157, and GH158.

**Figure 1:**
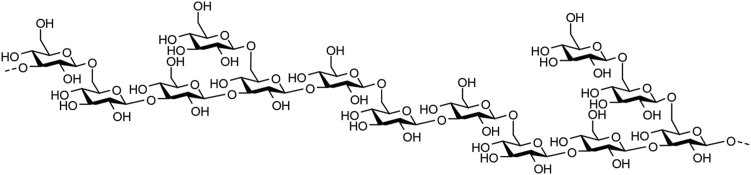
**Estimated chemical structure of laminarin from *Eisenia bicyclis*.** The chemical structure of laminarin was illustrated based on the report by Liu *et al.* (2).

GH55 contains bacterial and fungal inverting exo-β-1,3-glucosidase (EC 3.2.1.58), endo-β-1,3-glucanase (EC 3.2.1.39), and hesperidin 6-*O*-α-l-rhamnosyl-β-glucosidase (EC 3.2.1.168). Fungal GH55 β-1,3-glucanases are physiologically involved in conidial maturation and germination in *Aspergillus fumigatus* (6) and mycoparasitism in *Trichoderma harzianum* (7) through the hydrolysis of β1-3/1-6-glucans in the cell walls. Both exo-β-1,3-glucosidases and endo-β-1,3-glucanases degrade laminarin and are called as laminarinases. Laminarin is diverse β1-3/1-6-glucan derived from brown algae. Typical laminarin from *Eisenia bicyclis* consists of a β1-3- and β1-6-linked linear main chain and β1-6-linked branching d-glucosyl, gentiobiosyl, and gentiotriosyl residues (2). Endo-type enzymes degrade internal β1-3-linkages of the main chain of laminarin and produce more reducing sugars (oligosaccharide products) than d-glucose (7, 8, 9), but scissile linkages have not been clarified yet. Exo-β-1,3-glucosidases degrade β1-3-glucosidic linkages from nonreducing ends of the main chains even in the presence of branches, resulting in the liberation of gentiooligosaccharides (10, 11, 12, 13).

3D structures of GH55 exo-β-1,3-glucosidases have been determined so far in two fungal enzymes, *Phanerochaete chrysosporium* exo-β-1,3-glucosidase PcLam55A (13) and *Chaetomium thermophilum* CtLam55 (14), and a bacterial one, *Streptomyces* sp. SirexAA-E SacteLam55A (15). These enzymes share an overall structure resembling a rib cage, consisting of two β-helical domains (N- and C-domains) connected by a polypeptide linker. Both the β-helical domains contain 12 coils, each of which is composed of three β-strands and three loops (the *n*^th^ loop of *m*^th^ coil is called as C*m*-L*n*). The active site is located at the interface of the N- and C-domains. The Glu residue (equivalent to Glu-502 of SacteLam55A) in C8-L3 of the C-domain serves as a general acid catalyst with the help of the adjacent Tyr residue (Tyr-505 of SacteLam55A) in the orientation by a hydrogen bond. The proposed proton relay network of four amino acid residues (Thr-149, Ser-198, Gln-174, and Glu-480 in SacteLam55A) activates a water molecule, which attacks the anomeric carbon of the substrate from the other side of the scissile β-glucosidic bond (15). For the exo-type reaction by the exo-β-1,3-glucosidases, the nonreducing end d-glucosyl group of laminarioligosaccharide substrates interacts extensively in subsite −1 with Thr-149, Trp-446, Asp-449, Glu-480, and His-481 of SacteLam55A (15). In addition, the aromatic block, composed of the three conserved aromatic residues (Phe152, Trp444, and Trp446 in SacteLam55A), caps the substrate-binding cleft, resulting in the formation of the dead-end structure of subsite −1 (15). Furthermore, in the structures of fungal exo-β-1,3-glucosidases, a potential pocket accommodating the 6-*O*-linked branch moiety has been found in the proximity of subsite −1 (13, 14). This is essential for the successive hydrolysis of β1-3-linkage of β1-3/1-6-glucans from their nonreducing ends. Exo-β-1,3-glucosidases show high preference for long-chain laminarioligosaccharides (11, 13, 14) and have an extended substrate-binding cleft with six or more subsites at the domain interface (13, 14, 15). In the cleft of SacteLam55A, two aromatic residues, Tyr-194 and Trp-196, provide stacking interactions that are important for the high preference for long-chain substrates, although they are not conserved in GH55 enzymes (13).

Despite the progress made in understanding exo-acting enzymes on the basis of protein structure, the mechanism of the endo-acting mode of endo-β-1,3-glucanases on laminarin remains ambiguous. It is noteworthy that the sequence similarity suggests that endo-β-1,3-glucanases share with the exo-acting β-1,3-glucosidases not only the catalytic residues but also amino acid residues forming subsite −1 and the aromatic block (Fig. 2).

**Figure 2:**
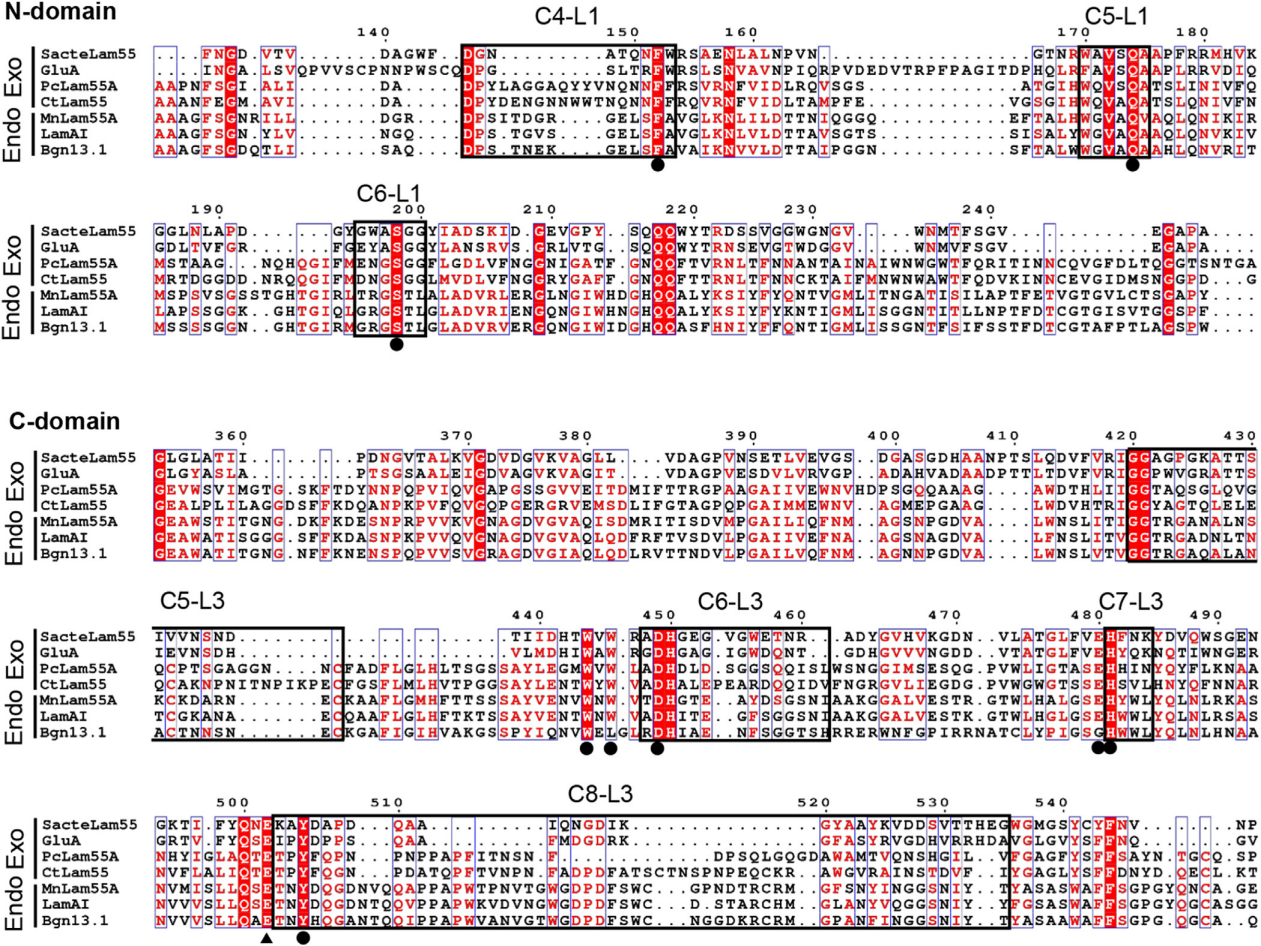
**Comparison of partial sequences of characterized GH55 enzymes.** The amino acid sequences were aligned with MAFFT ver. 7 (35). The result of alignment was visualized using ESPript 3.0 (36). *Closed circles* indicate highly conserved residues in GH55 enzymes; a *closed triangle* indicates general acid catalyst; *squares with thick borders* indicate the loops. GH55, glycoside hydrolase family 55.

In this study, the endo-wise action of GH55 endo-β-1,3-glucanase was investigated. MnLam55A was identified from extracellular proteins of a phytopathogenic fungus *Microdochium nivale*, which infects grasses and cereals and causes pink snow mold (16, 17). The recombinant MnLam55A catalyzed endo-acting hydrolysis by initially cleaving the nonreducing end of laminarioligosaccharide moieties adjacent to β1-6 linkages in the laminarin main chain. Through protein structure determination together with structural comparison and the docking analysis, we found that this enzyme had the subsite −2 structure suitable for the binding of a β1-6-linked d-glucosyl group and the extended cleft that is expected to form minus subsites. The high conservation of the associated residues in GH55 endo-β-1,3-glucanases also supports the idea that these structures are essential for the endo-acting hydrolysis.

## Results

### Identification of MnLam55A

**Figure 3:**
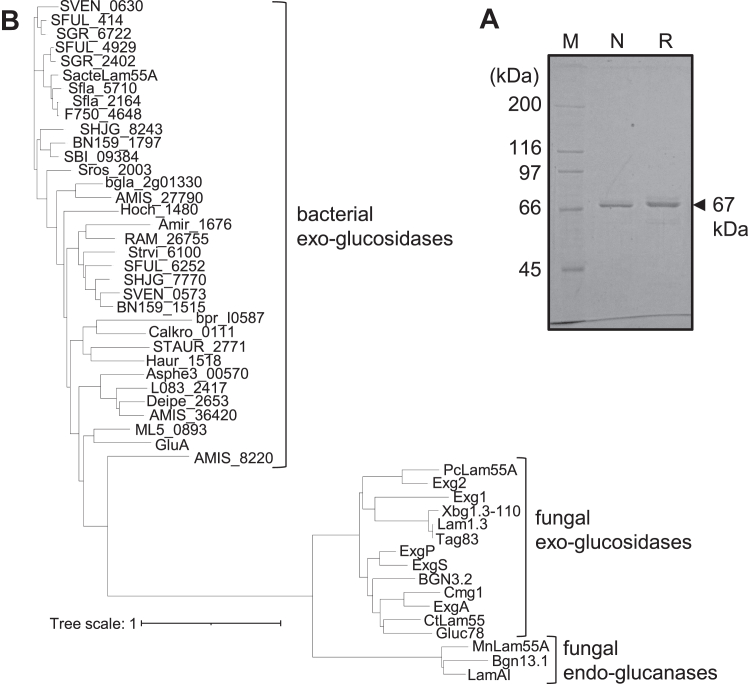
**Identification of MnLam55A.***A*, SDS-PAGE of native and recombinant MnLam55A. Lane M, protein markers; lane N, native MnLam55A; lane R, recombinant MnLam55A. *B*, phylogenic tree of characterized GH55 enzymes was made by MAFFT ver. 7 (35) and visualized by iTOL ver. 6 (37). GH55, glycoside hydrolase family 55.

MnLam55A (0.182 mg, specific activity of 16.8 U/mg) was purified to homogeneity from the culture supernatant of *M. nivale*, cultured for 4 weeks at 18 °C. The purified enzyme showed a single band of 68 kDa on SDS-PAGE (Fig. 3*A*). The tryptic digests of MnLam55A were subjected to LC-MS/MS analysis to identify the *MnLam55A* gene from the *M. nivale* genome (18). The spectra obtained by tandem mass spectrometry of the tryptic digests matched the deduced sequence with 47% coverage of the entire sequence of the putative GH55 protein (DDBJ accession number: LC773407). The N-terminal 19 residues (Met to Ala) in the deduced sequence were a putative cleavable signal sequence, and the next Ser (Ser-1) was the N-terminal residue of the mature protein. A Web BLASTp search of MnLam55A against nonredundant protein sequences showed the highest identity at 87% to glucan endo-1,3-β-glucosidase BGN13.1 from *Microdochium bolleyi* (NCBI ID: KXJ95237.1). Among characterized GH55 enzymes, the highest identity at 64% was found for LamAI from *Trichoderma viride* U-1 (8, 9). In the phylogenetic analysis of characterized GH55 enzymes, MnLam55A fell into a clade of fungal endo-β-1,3-glucanases together with LamAI (Fig. 3*B*). MnLam55A possesses all of the highly conserved residues of GH55 enzymes for the formation of the catalytic site and subsite −1 as follows: the general acid catalyst, Glu-614; its adjusting residue, Tyr-617; the proton relay network-activating substrate water, Glu-140, Ser-203, Gln-172, and Glu-591; the subsite −1 forming residues, Glu-140, Trp-552, Asp-557, Glu-591, and His-592; and the aromatic block, Phe-143, Trp-552, and Trp-554 (the residue numbers are from N-terminal Ser-1 of mature MnLam55A) (Fig. 2).

Recombinant MnLam55A was extracellularly produced in a *Komagataella pastoris* transformant harboring the gene encoding MnLam55A with the N-terminal signal sequence of α-factor replacing the original signal sequence. In 1.2 l of the culture broth, 491 U of laminarinase activity was produced after 96 h of cultivation. Purified recombinant MnLam55A (5.57 mg, 94.4 U) showed 16.9 U/mg of specific activity and a single band of 67 kDa on SDS-PAGE, consistent with those of the native enzyme (Fig. 3*A*). The apparent molecular mass on SDS-PAGE was lower than the calculated mass (80 kDa) from amino acid sequences. This smaller apparent molecular mass on SDS-PAGE was also reported in other fungal GH55 endo-β-1,3-glucanases BGN13.1 from *T. harzianum* (7) and lamAI from *T. viride* (9) and is understood as anomalous migration due to the strong affinity to the gel matrix. Recombinant MnLam55A showed the highest activity at pH 5.6 and 50 °C (Fig. S1, *A* and *B*). This enzyme retained ≥95% of the original activity after incubation in a pH range of 4.3 to 9.3 (4 °C, 24 h) and at ≤45 °C (for 20 min) (Fig. S1, *A* and *B*).

### Time course of laminarin hydrolysis by MnLam55A

**Figure 4:**
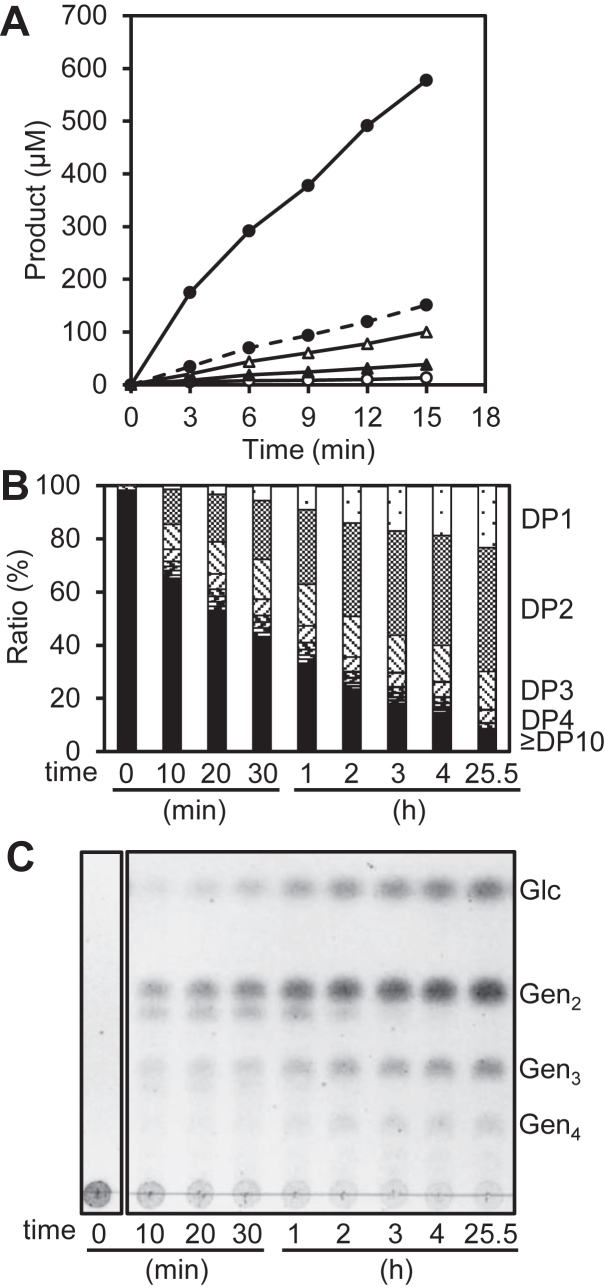
**Progress of reaction product formation from laminarin by MnLam55A.***A*, time course of increase in initial reaction products from 4 mg/ml laminarin. *Solid line with closed circles* shows the concentrations of reducing sugars, and *broken line with closed circles* shows the concentrations of the sum of d-glucose, gentiobiose, and gentiotriose. *Open circles*, *closed arrowheads*, and *open arrowheads* show d-glucose, gentiotriose, and gentiobiose, respectively. *B*, time course of degree of polymerization (DP) of the products from 10 mg/ml laminarin. Percentage was based on the weight ratio. *C*, TLC of the same samples shown in *panel B*. Gen_*n*_, gentiooligosaccharide of DP*n*; Glc, glucose.

The reaction products from laminarin were monitored. At the early stage of the reaction, total molar concentration of reducing sugars was 4-fold higher than the sum of those of d-glucose, gentiobiose, and gentiotriose, which were possibly released from nonreducing end of laminarin through exo-acting hydrolysis (Fig. 4*A*). This result suggests that MnLam55A has both endo- and exo-acting activities on laminarin but acts it mainly in an endo-acting manner. Using 62.5-fold higher concentration of enzyme, the reaction approached the complete digestion of laminarin (Fig. 4, *B* and *C*). d-Glucose and oligosaccharides with degrees of polymerization (DPs) of 2 to 4 gradually accumulated with decrease of laminarin (DP ≥10). After 25.5 h of reaction, 90% of laminarin was degraded to monosaccharide and oligosaccharides of DP2–4 (Fig. 4*B*). The disaccharide, one of the complete degradation products accumulated in the reaction, migrated similarly to the gentiobiose standard in the TLC analysis. The trisaccharide and tetrasaccharide were isolated by gel-filtration column chromatography, and their chemical structures were analyzed by electrospray ionization (ESI)-mass spectrometry (MS) and NMR. In MS spectra of the trisaccharide and the tetrasaccharide, [M+Na]^+^ ion peaks of *m/z* 527.16 and 689.21 were detected, respectively. From the NMR analysis, the chemical shifts from ^13^C NMR spectra of the trisaccharide and the tetrasaccharide matched those of gentiotriose (19) and gentiotetraose (20), respectively. MnLam55A catalyzed hydrolysis of laminarin mainly in the endo-acting manner at the early stage of the reaction and almost completely hydrolyzed laminarin to d-glucose and gentiooligosaccharides (DP2–4) through its exo-acting activity.

### Determination of initially cleaved linkage of laminarin

**Figure 5:**
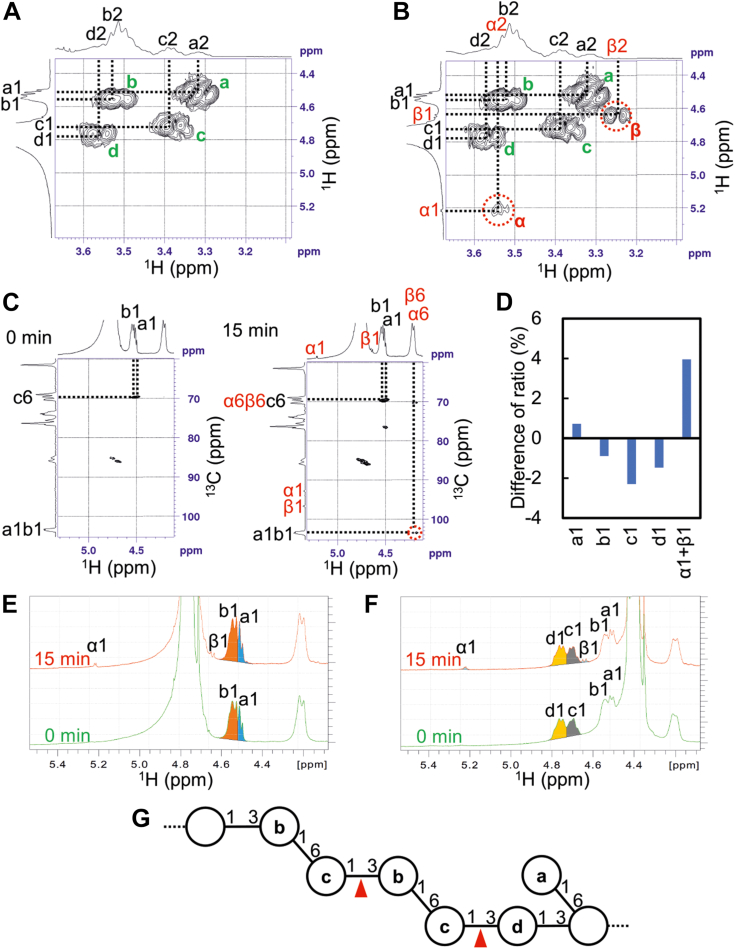
**NMR analysis of initial reaction products of MnLam55A with laminarin.***A*, COSY 2D-NMR spectrum of reaction mixture at 0 min of Figure 4*A*. Correlation peaks indicated as a–d are corresponded to labeled d-glucosyl residues in the model figure of laminarin. *B*, COSY 2D-NMR spectrum of reaction mixture at 15 min of Figure 4*A*. *C*, HMBC 2D-NMR spectra of reaction mixture at 0 and 15 min of Figure 4*A*. *D*, the difference of integration ratio of 1-H proton signals colored in (*E* and *F*). *E* and *F*, ^1^H-NMR spectra of reaction mixture at 0 and 15 min shown in Figure 4*A* recorded at 27 °C (*E*) and 60 °C (*F*). *G*, the determined endo-acting cleavage sites in laminarin by MnLam55A. HMBC, heteronuclear multiple bond correlation.

In order to determine the initial cleavage site of laminarin, NMR analysis of early reaction products from laminarin was performed. COSY 2D-NMR spectrum of laminarin showed four correlation peaks between 1-H and 2-H, which corresponded to the four d-glucosyl residues: a, Glc1→6; b, →3Glc1→6; c, →6Glc1→3; and d, →3Glc1→3 (a–d shown in Fig. 5*A*). In addition to these peaks, two correlation peaks derived from the newly produced residues (α and β) were observed in the COSY 2D-NMR spectrum of the 15-min reaction product (Fig. 5*B*). The heteronuclear multiple bond correlation 2D-NMR spectra of this reaction product showed the clear correlation peak of 1-C from a and b and 6-H from α and β (*δ*_H_ 4.19→*δ*_C_ 103.4), although those of 1-H from a and b and 6-C from sugar residues α and β (*δ*_H_ 4.50→*δ*_C_ 69.4) were covered by other peaks (Fig. 5*C*). The correlation peaks of 3-C from α and β and proton signals from any other d-glucosyl residues were not observed (Fig. 5*C*). These results indicated that α and β were 6-*O*-β-glycosylated d-glucose residues at the reducing end (→6Glc).

Differences of the integration ratio to the sum of the integrations of each 1-H signal between 0- and 15-min reaction solutions were calculated (Fig. 5*D*). The 1-H signals of a and b were obtained by ^1^H NMR analysis at 27 °C, while those of c and d were conducted at 60 °C to avoid overlapping the large signal of H_2_O (Fig. 5, *E* and *F*, respectively). Anomeric proton signals of sugar residues, a–d, are shown as a1–d1, respectively, and 1-H signals of the reducing end residue of the reaction products are shown as α1 and β1. The ratio of c1 decreased the most, and it was followed by d1 and b1 (Fig. 5*D*). The signal of a1 slightly increased and that of α1+β1 significantly increased. The sum of the decreasing ratio of b1 and d1 was almost equal to that of c1, and the increasing ratio of a1 was almost equal to the decreasing ratio of b1. These results indicated that MnLam55A cleaved the β1-3-linkages between sugar residues c and b/d (nonreducing end 6-*O*-glycosylated β1-3-glucosidic linkage of β1-3-linked laminarioligosaccharide portions of laminarin) and produced the oligosaccharides harboring 6-*O*-β-glycosylated d-glucose residues at reducing end (α and β) (Fig. 5*G*).

### Reaction products from laminarioligosaccharides

**Figure 6:**
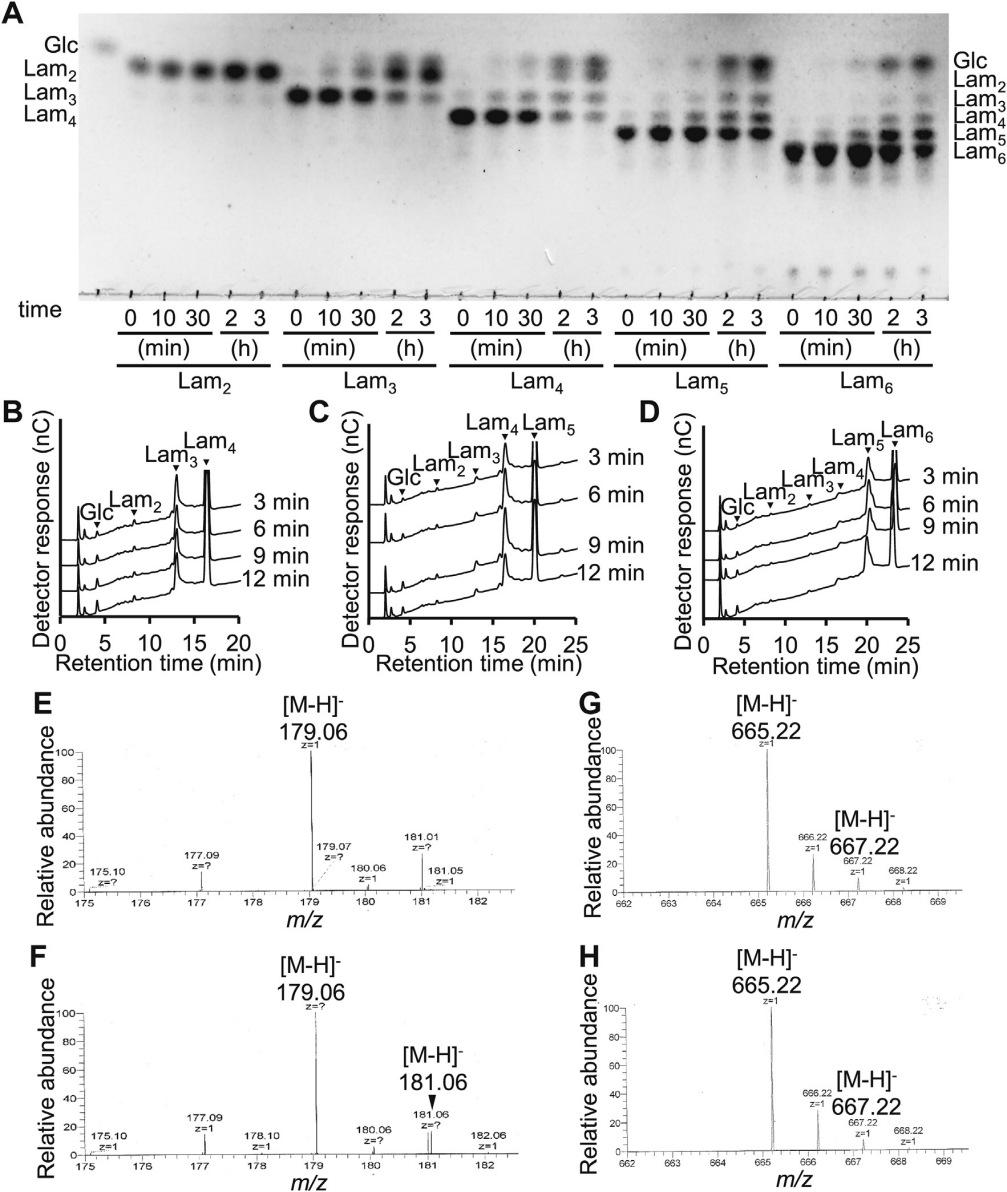
**Reaction of MnLam55A with laminarioligosaccharide.***A*, TLC analysis of MnLam55A reaction products. MnLam55A (17.4 nM) was incubated with 10 mM laminarioligosaccharides (DP2–6). Lam_*n*_, laminarioligosaccharide of DP*n*. *B–D*, high-performance anion-exchange chromatography with pulsed amperometric detection analysis of MnLam55A initial products. MnLam55A (17.4 nM) was incubated with 10 mM laminaritetraose (*B*), laminaripentaose (*C*), or laminarihexaose (*D*). *E*–*H*, ESI-MS spectra of reaction products from 2 mM laminaripentaose in H_2_O or H_2_^18^O. The mass peak of *m*/*z* 181.01 in both (*E* and *F*) is from a contaminating compound. DP, degree of polymerization; ESI, electrospray ionization; MS, mass spectrometry.

Reactions of MnLam55A with laminarioligosaccharides (DP2–6) were analyzed. MnLam55A degraded laminaritriose and longer laminarioligosaccharides, but not laminaribiose (Fig. 6*A*). At the early stage of the reactions with laminarioligosaccharides (DP4–6), d-glucose was most commonly produced (Fig. 6, *B*–*D*), indicating the dominant exo-type hydrolysis of laminarioligosaccharides. To identify the terminal of the substrates releasing d-glucose, MS analysis of the products from laminaripentaose hydrolyzed in H_2_O and H_2_^18^O was performed. A mass peak of *m/z* 181.06, corresponding to [M−H]^−^ of ^18^O-containing d-glucose, was observed only in the spectrum of the reaction with H_2_^18^O (Fig. 6, *E* and *F*). In contrast, the mass peak corresponding to [M−H]^−^ of ^18^O-containing laminaritetraose was not observed. The weak mass signal at *m/z* 667.22, two units higher than that of laminaritetraose, was observed in both the reactions with H_2_O and H_2_^18^O and was considered to be the natural isotope of laminaritetraose (Fig. 6, *G* and *H*).

### Substrate specificity of MnLam55A

Reaction velocities for various β-glucans and laminarioligosaccharides (DP2–6) were measured. MnLam55A acted on laminarin (44.8 ± 6.3 s^−1^), but not on scleroglucan (β1-3/1-6-glucan, β1-3-linked main chain with β1-6-d-glucosyl branches), curdlan (liner β1-3-glucan), paramylon (liner β1-3-glucan), and barley β1-3/1-4-glucan (<0.110 s^−1^). MnLam55A showed 1400-fold higher *k*_cat_/*K*_m_ to laminarin (5920 s^−1^ mM^−1^) than even to laminarioligosaccharides of DP 3, which showed the highest *k*_cat_/*K*_m_ (4.21 s^−1^ mM^−1^) among the laminarioligosaccharides (Table 1).

**Table:** Kinetic parameters of recombinant MnLam55A

Substrate	*k*_cat_ (s^−1^)	*K*_m_ (mM)	*k*_cat_/*K*_m_ (s^−1^ mM^−1^)	Relative *k*_cat_/*K*_m_
Laminaritriose	24.3 ± 2.4	5.86 ± 1.03	4.21	7.11 × 10^−4^
Laminaritetraose	17.4 ± 1.3	4.68 ± 0.89	3.79	6.40 × 10^−4^
Laminaripentaose	13.1 ± 1.5	5.02 ± 1.07	2.65	4.48 × 10^−4^
Laminarihexaose	n.a.	n.a.	0.497	8.40 × 10^−5^
Laminarin	56.1 ± 5.2	0.00947 ± 0.00162	5920	1

Abbreviation: n.a., not analyzed.

### 3D structure of MnLam55A

**Figure 7:**
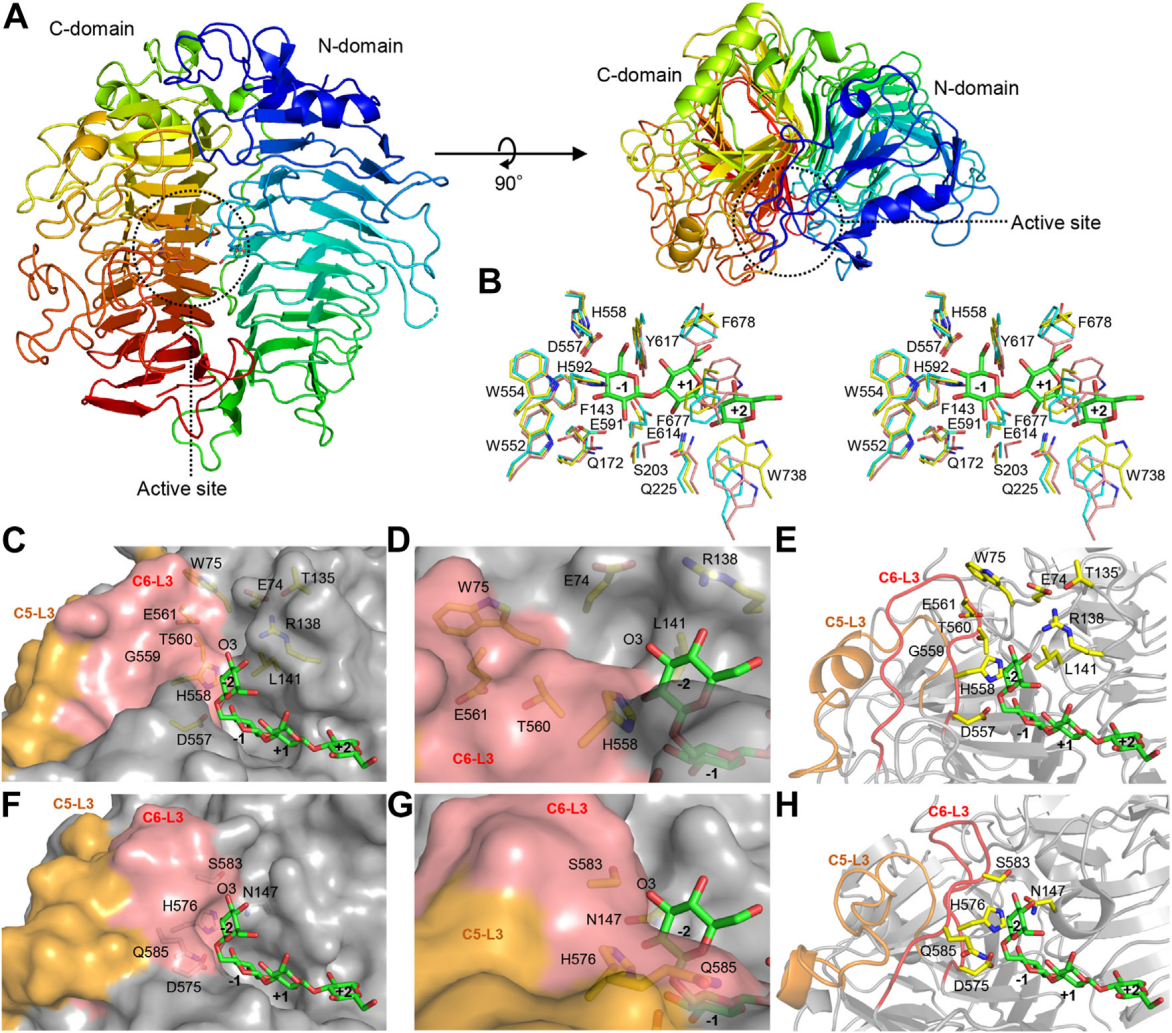
**3D structure of MnLam55A.***A*, the crystal structure of MnLam55A was determined in unliganded form at 2.4 Å resolution. *B*, stereo view of the structure around subsites −1 to +2. The residues of MnLam55A (*yellow*), PcLam55A (PDB ID: 3EQO; *cyan*), and SacteLam55A (PDB ID: 4TZ1; *pink*) and laminaritriose from 4TZ1 were shown. *C*–*E*, 6^Ⅲ^-*O*-glucosyl laminaritriose docked MnLam55A structure using AutoDock Vina. *F*–*H*, the corresponding structure of PcLam55A. The ligand is from the docking model of MnLam55A.

The crystal structure of MnLam55A was determined in unliganded form at 2.4 Å resolution. The solved structure contains two molecules of MnLam55A in the asymmetric unit. The entire structure consists of two β-helical domains of 3-solenoid connected by a linker (Fig. 7*A* and Fig. S2). Each of the domains is composed of 12 coils, which each contains three strands and three loops as observed in GH55 exo-β-1,3-glucosidases (Fig. 7*A*). RMSD for Cα atoms of MnLam55A and GH55 exo-β-1,3-glucosidases is 1.38 Å to PcLam55A (PDB ID: 3EQO), 1.25 Å to CtLam55 (PDB ID: 5M60), and 2.79 Å to SacteLam55A (PDB ID: 4PEW). Thus, the whole structure of MnLam55A is more similar to the fungal enzymes than to the bacterial one. The active site and substrate-binding site of MnLam55A were compared with those of PcLam55A (PDB ID: 3EQO) and SacteLam55A (PDB ID: 4TZ1) (Fig. 7*B*). In MnLam55A, the catalytic site and subsite −1 are formed by Glu-140, Gln-172, Ser-203, Trp-552, Asp-557, Glu-591, His-592, Glu-614, and Tyr-617, the aromatic block is formed by Phe-143, Trp-552, and Trp-554, and subsite +1 is formed by Gln-225, Tyr-617, Phe-677, and Phe-678. These residues are spatially conserved well with those of exo-β-1,3-glucosidases (Fig. 7*B*). Residues forming subsite +2 of MnLam55A are Gln-225 and Trp-738, which are spatially placed at similar positions of the corresponding residues of exo-β-1,3-glucosidases, although their orientation is less conserved compared to the residues at subsites −1 and +1 (Fig. 7*B*). As the plus subsites for the binding of longer oligosaccharides, the aromatic residues suggested in SacteLam55A (Trp-196 at subsite +2 and Tyr-194 at subsite +5) and those on the putative substrate binding groove in PcLam55A (Tyr-135, Phe-199, and Trp-245) are not observed in MnLam55A. Docking simulation of MnLam55A with 6^Ⅲ^-*O*-glucosyl laminaritriose was performed to predict a binding mode of the nonreducing end β1-6-linked Glc^Ⅳ^ group to the enzyme. In the docked model, Glc^Ⅲ^ of the ligand was accommodated at subsite −1, where the nonreducing end d-glucosyl residue of laminaritriose was predicted as shown in Figure 7*B* (Fig. 7*C*). The Glc^Ⅳ^ residue was accommodated in possible subsite −2, corresponding to the expected space in the fungal GH55 exo-β-1,3-glucosidases (13, 14). His-558 was suitably located to interact with Glc^Ⅳ^ in subsite −2 (Fig. 7, *D* and *E*). In the structure of PcLam55A (PDB ID: 3EQO), the corresponding His-576 was similarly located but more residues seemed to be involved in the formation of the pocket-shaped subsite −2 (Fig. 7, *F*–*H*). In contrast, MnLam55A possesses no equivalent residues, particularly to Ser-583, mainly due to the differentially oriented C6-L3. The 3-hydroxy group of Glc^Ⅳ^ in the MnLam55A structure was located in an open space on an extended cleft structure (Fig. 7, *C* and *D*). This cleft is formed at the interface of the two domains: C6-L3 in the C-domain (main chain of Gly-559, Thr-560, and Glu-561) to one side, and C4-L1 and C2-L1 of the N-domain involving Leu-141, Arg-138, Thr-135, Glu-74, and Trp-75 for the other (Fig. 7*E*). The extended cleft was expected to form the minus subsites beyond subsite −2.

## Discussion

β-1,3-Glucanases are distributed among various organisms and degrade β1-3-glucans with various structures (21). GH55 contains exo-β-1,3-glucosidases and endo-β-1,3-glucanases acting on laminarin (5). The dead-end structure of subsite −1 is regarded to be indispensable for the exo-type reaction of exo-β-1,3-glucosidases (13, 14, 15), but the sequence comparison suggests that the residues are conserved in endo-acting enzymes in this family (7, 8, 9). In this study, the mechanism for laminarin degradation by GH55 endo-β-1,3-glucanase was investigated using MnLam55A. Firstly, the cleavage site in laminarin was determined by functional analyses. MnLam55A has highly similar amino acid sequences to fungal GH55 endo-β-1,3-glucanases (Figs. 2 and 3*B*) and exhibited endo-type action in the reaction with laminarin (Fig. 4*A*) but no activity with β1-3-glucans containing no β1-6-linkages in the main chain, whereas typical endo-β-1,3-glucanases in families other than GH55 randomly degrade the β1-3-glucans (22, 23). This indicates that β1-6-linkages in the main chain of laminarin are essential for the hydrolysis by MnLam55A, and MnLam55A is clearly discriminated from the typical endo-β-1,3-glucanases. NMR analysis of the initial products from laminarin showed that MnLam55A predominantly hydrolyzed β1-3-linkages at the nonreducing end of the laminarioligosaccharide moiety adjacent to β1-6 linkage in the laminarin main chain and produced products containing gentiobiose residues at the reducing end (Fig. 5*G*). As this enzyme almost completely degraded laminarin to d-glucose and gentiooligosaccharides (Fig. 4) and showed exo-type action in the reaction with laminarioligosaccharides (Fig. 6), this suggested that laminarin was cleaved first by the endo-acting hydrolysis, followed by successive exo-acting hydrolysis of every β1-3-linkage regardless of the presence of β1-6-linked branching moieties from the nonreducing ends, as observed in GH55 exo-β-1,3-glucosidases (10, 11, 12, 13). The lack of remaining 3-*O*-β-d-glucosyl gentiooligosaccharides after complete degradation of laminarin (Fig. 4*C*) suggested that MnLam55A hydrolyzes the last reducing-end β1-3-d-glucosidic linkage when linked to gentiooligosaccharides, although laminaribiose is not a substrate of this enzyme. MnLam55A acted similarly on nonreducing terminal β1-3-d-glucosidic linkages of laminarioligosaccharides and laminarioligosaccharide moieties of laminarin but with very different *k*_cat_/*K*_m_ values (Table 1). Laminarin is much preferred over the oligosaccharides, even though the longer oligosaccharides showed lower *k*_cat_/*K*_m_. These results suggest that the long nonreducing end part of laminarin from the scissile β1-3-d-glucosidic linkage provides high-binding affinity. Beyond subsites −1 and −2 which accommodated β1-6-linked gentiobiose moieties, further minus subsites were expected for binding the β1-3/1-6-glucan of laminarin main chain in the hydrolysis by MnLam55A.

**Figure 8:**
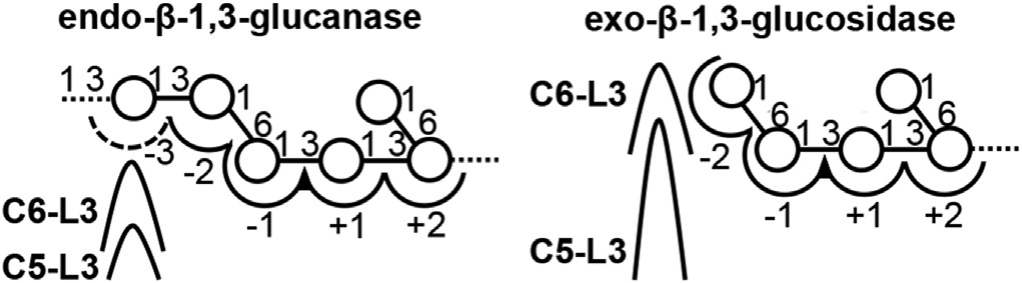
**Substrate-binding mode of GH55 enzymes.** Predicted substrate-binding mode of exo-β-1,3-glucosidases and endo-β-1,3-glucanases. GH55, glycoside hydrolase family 55.

This work provides the first ternary structure of endo-acting GH55 β-1,3-glucanases. The structure indicates that MnLam55A shares with the GH55 exo-β-1,3-glucosidases (13, 14, 15) not only overall structure but also the residues forming the catalytic site and subsite −1 including the aromatic block (Fig. 7*B*). The exo-type action of MnLam55A on laminarioligosaccharides and laminarioligosaccharide moieties in the main chain of laminarin is attributed to the structure for exo-acting enzymes. The possible subsite −2 structure of MnLam55A was probed by the docking study with 6^Ⅲ^-*O*-d-glucosyl laminaritriose (Fig. 7*C*). The space accommodating the nonreducing end d-glucosyl residue (Glc^Ⅳ^) was equivalent to the pocket structure in fungal GH55 exo-β-1,3-glucosidases for binding of the β1-6-branching moiety of substrate (13, 14), whereas the corresponding space was not present in the structure of bacterial GH55 exo-β-1,3-glucosidase SacteLam55A (15). The possible binding residue in subsite −2, His-558 of MnLam55A, is conserved well in the fungal GH55 exo-β-1,3-glucosidases and endo-β-1,3-glucanases (Figs. 2 and 7, *C*–*H*). PcLam55A, however, additionally has Asn-147, Ser-583, and Gln-585 involved in the formation of the closed pocket structure, preventing 3-*O*-glycosylated d-glucosyl group from binding to subsite −2 (Fig. 7, *F*–*H*). Asn-147 and Gln-585 are highly conserved in exo-type enzymes, but endo-type enzymes including MnLam55A share no corresponding residues (Fig. 2). On the contrary, MnLam55A has an open cleft, extending from subsite −2 toward the nonreducing part-binding site, to place 3-hydroxy group of the d-glucosyl residue in subsite −2 toward an open space on the cleft in the docking model (Fig. 7*D*). Thus, this cleft possibly forms minus subsites beyond subsite −2 to accommodate the laminarin main chain linked to 3-O of the d-glucosyl residue bound to subsite −2. The cleft-forming residues of N-domain, Trp-75, Thr-135, and Leu-141 of MnLam55A, are conserved well in other endo-β-1,3-glucanase sequences, which suggests their importance for the formation of minus subsites. Another structure directly involved in the formation of this cleft in MnLam55A is C6-L3 of the C-domain, whereas the corresponding loop of GH55 exo-β-1,3-glucosidases are differentially located close to subsite −2 to form the closed pocket-shaped structure (Fig. 7, *C*–*H*). The difference in the C6-L3 localization is presumably caused by the structural difference of the adjacent C5-L3 (Fig. 7, *C*–*H*). C5-L3 of MnLam55A is shorter than the corresponding loops of fungal exo-type enzymes (Fig. 2). The smaller size of C5-L3 in the structure probably allows the localization of C6-L3 appropriately for the formation of the extended cleft and possible subsites (illustrated in Fig. 8). In addition to the conservation of the cleft-forming residues in primary structures, the comparable lengths of the C5-L3 loops in fungal endo-β-1,3-glucanases suggest that the extended cleft possibly involved in the formation of minus subsites, observed in MnLam55A, is shared by GH55 endo-β-1,3-glucanases (Fig. 2). It is noteworthy that the bacterial exo-acting enzymes such as SacteLam55A possess even shorter C5-L3 than MnLam55A (Fig. 2), but SacteLam55A has no space for subsite −2 due to the occupation of the space by Phe-143, Trp-144, and Gln-150 in the ternary structure of SacteLam55A.

The endo-acting MnLam55A, like other GH55 endo-β-1,3-glucanases, showed the greatest preference for laminaritriose among laminarioligosaccharides (Table 1), whereas GH55 exo-β-1,3-glucosidases prefer the longer ones (11, 13). All of them commonly show very low or undetectable activity to laminaribiose (Fig. 6*A*) (9, 11, 13, 14, 15). The trisaccharide requirement for activity commonly observed in the GH55 enzymes is probably due to the well-conserved structure of subsites −1 and +1, with a little difference in the subsite +2-forming residues (Figs. 2 and 7*B*). Laminaritriose is probably required for productive binding. In contrast, some aromatic residues such as Trp-196 and Tyr-194 in SacteLam55A at subsites +2 and +5, respectively (15), and Tyr-135, Phe-199, and Trp-245 on the long groove in PcLam55A (13) are involved in the formation of the plus subsites, but MnLam55A lacks these aromatic residues. This probably causes the different preference for long-chain laminarioligosaccharides.

In conclusion, we found that endo-acting hydrolysis of MnLam55A cleaves the β1-3-linkages at the nonreducing end of the laminarioligosaccharide moiety adjacent to β1-6-linkages in the laminarin main chain with significantly higher affinity than exo-wise hydrolysis of laminarioligosaccharides. Crystal structure determination and the docking analysis revealed that MnLam55A shares with fungal GH55 exo-β-1,3-glucosidases overall structures and subsites −2 to +2, including the binding mode of the gentiobiose moiety in subsites −1 and −2, but the possible substrate-binding cleft extending from subsite −2 was found in MnLam55A structure for the first time. The relevant structures are similar in other GH55 endo-β-1,3-glucanases. Based on these findings, we present the substrate binding modes in GH55 endo-acting β-1,3-glucanases (Fig. 8).

## Experimental procedures

### Identification of MnLam55A from the culture supernatant of *M. nivale*

*M. nivale* strain MCW222-7 was originally obtained from wheat in Memuro, Hokkaido, Japan and stored at −20 °C. It was grown on potato dextrose agar medium (Becton, Dickinson and Company) and cultured in potato dextrose broth (0.8 l) following previous work (18). Culture supernatant (0.7 l), collected by centrifugation (6000×*g*, 10 min, 4 °C), was loaded on a DEAE Sepharose Fast Flow column (Cytiva; 2.8 cm I.D. × 25 cm) equilibrated with 10 mM sodium phosphate buffer (pH 7.0). Nonadsorbed protein, eluted by the same buffer, was separated on a Butyl Sepharose Fast Flow 4 column (Cytiva; 2.8 cm I.D. × 16 cm) equilibrated with 10 mM sodium phosphate buffer (pH 7.0) containing 1.2 M ammonium sulfate. After washing the column with the same buffer, adsorbed protein was eluted with a linear gradient of 1.2 to 0 M ammonium sulfate. Active fractions were concentrated by ultrafiltration using Amicon Ultra YM30 (molecular weight cut-off, 30,000; Merck Millipore), loaded on a Sephacryl S-200 column (Cytiva; 1.5 cm I.D. × 98 cm), and equilibrated with 10 mM sodium phosphate buffer (pH 7.0) containing 0.1 M NaCl. Active fractions were collected, and 2.4 μg of the purified enzyme was further separated by SDS-PAGE. The single-protein band of MnLam55A was cut out and in-gel tryptic digest was performed using In-Gel Tryptic Digestion kit (Thermo Fisher Scientific). The resulting peptides, purified by the Pierce C18 Spin column (Thermo Fisher Scientific), were analyzed with LC-MS/MS Paradigm MS2 (Michrom BioResources) following previous research (18). Data were processed using the search software Proteome Discoverer (Thermo Fisher Scientific, https://www.thermofisher.com/jp/ja/home/industrial/mass-spectrometry/liquid-chromatography-mass-spectrometry-lc-ms/lc-ms-software/multi-omics-data-analysis/proteome-discoverer-software.html) with a draft genome sequence of *M. nivale* (18).

### Preparation of recombinant MnLam55A in *K. pastoris*

The complementary DNA of MnLam55A was amplified from a complementary DNA pool, prepared from total RNA of 4-week-cultured *M. nivale* cells (18), by PCR using primers (5′-ATGGTCAGACTCCCTGCCCT-3′, sense; and 5′-AGGGGTATATCTGCCGACAA -3′, antisense) and KOD FX DNA polymerase (Toyobo). The amplified DNA fragment was inserted into pPICZαA (Invitrogen; between α-factor signal sequence and *c-myc* epitope sequence) using In-Fusion HD Cloning Kit (Takara Bio). The cloned DNA was sequenced using an Applied Biosystems 3130 Genetic Analyzer (Applied Biosystems). This *Sac*Ⅰ-linearized plasmid was introduced into *K*. *pastoris* X-33 by electroporation. The transformant was cultured following published research (18), but using 600 ml of BMGY medium and 1.2 l of BMMY medium containing 2% methanol. During the 96-h incubation in BMMY, methanol (24 ml) was added every 24 h. MnLam55A was purified from the culture supernatant (1.1 l). Proteins were precipitated with 90% saturated ammonium sulfate, dissolved in 10 mM sodium phosphate buffer (pH 7.0, 140 ml), and separated with a Butyl Toyopearl 650 M column (Tosoh; 2.8 cm I.D. × 16 cm) as previously described (18). Active fractions were further separated by two DEAE Sepharose Fast Flow chromatographic columns; recombinant MnLam55A was eluted in nonadsorbed and adsorbed fractions in the first and second chromatographic runs, respectively. As starting buffers, 10 mM sodium phosphate buffer (pH 7.0) and 10 mM glycine–NaOH buffer (pH 9.0) were used in the first and second columns, respectively. Elution of adsorbed protein in the second run was performed with a linear gradient of 0 to 0.5 M NaCl. The active fraction was dialyzed against 10 mM glycine–NaOH buffer (pH 9.0), concentrated as described above, and stored at 4 °C until analysis. Protein concentration of the purified enzyme was determined by amino acid analysis using a high-speed amino acid analyzer L-8900 (Hitachi) after complete hydrolysis in 6 M HCl at 110 °C for 24 h.

### Standard enzyme assay

Production of reducing sugar from laminarin from *E*. *bicyclis* (Nacalai Tesque) was measured. A reaction mixture (100 μl) consisting of 4 mg/ml laminarin, 40 mM sodium acetate buffer (pH 5.1), 0.2 mg/ml bovine serum albumin (BSA), and appropriate concentration of enzyme was incubated at 30 °C for 12 min. Reaction mixture (10 μl) was taken every 3 min, and reducing sugar was quantified by measuring *A*_560_ according to the copper-bicinchoninate method (24). One U of enzyme activity was defined as enzyme amount that produces 1 μmol of reducing sugar equivalent to d-glucose in 1 min under these conditions.

### Effect of pH and temperature

The optimum pH was determined from reaction rates at various pH values. The reaction solutions were prepared as in the standard assay, but 80 mM Britton–Robinson buffer (pH 3.5–7.0; a mixture of phosphate, acetic acid, and glycine (80 mM each) titrated with 0.5 M NaOH) was used. The stable pH range was determined by residual activity after incubation of 348 nM MnLam55A in 20 mM Britton–Robinson buffer (pH 2.5–12.0) at 4 °C for 24 h. The optimum temperature was determined by the reaction rates in the standard assay but 10 to 70 °C. The stable range of MnLam55A against temperature was determined from residual activity after incubation of 174 nM MnLam55A in 67 mM sodium acetate buffer (pH 5.1) at 30 to 65 °C for 20 min. The range in which the enzyme retained ≥95% of the original activity was regarded as stable range. Three independent replications of each experiment were conducted.

### Time course of the reaction with laminarin

To analyze the initial products, the reaction with laminarin was performed using a low concentration of the enzyme for a short period. A reaction mixture consisting of 4 mg/ml laminarin, 40 mM sodium acetate buffer (pH 5.6), 0.2 mg/ml BSA, and 13.9 nM MnLam55A, was incubated at 30 °C for 15 min. The reaction was terminated by heating at 100 °C for 3 min. Reducing sugar was quantified as described above, and sugar content was analyzed by high-performance anion-exchange chromatography with pulsed amperometric detection under following conditions: column, CarboPac PA-1 (Thermo Fisher Scientific; 4 mm I.D. × 250 mm); elution, linear gradient of 0 to 250 mM sodium acetate in 0.2 M NaOH; flow rate, 0.8 ml/min; and detection, pulsed amperometry. For NMR analysis, the reaction solutions (740 μl) at 0 and 15 min were lyophilized and dissolved in D_2_O. ^1^H NMR, COSY 2D-NMR, ^13^C NMR, heteronuclear single quantum coherence 2D-NMR, and heteronuclear multiple bond correlation 2D-NMR spectra were measured using Avance Neo (Bruker). Data were analyzed using TopSpin version 3.6.2 (Bruker, https://www.bruker.com/ja/products-and-solutions/mr/nmr-software/topspin.html).

The progress of the reactions was monitored as follows. A reaction mixture of 10 mg/ml laminarin, 11 mM sodium acetate buffer (pH 5.6), 0.05 mg/ml BSA, and 870 nM MnLam55A was incubated at 30 °C for 25.5 h. Aliquots (100 μl) taken at several timepoints were heated at 100 °C for 3 min. Sugar content of the samples was analyzed by TLC using a Silica gel 60 F_254_ plate (Merck) developed with 2-propanol:1-butanol:water = 12:3:4 (v/v/v) and HPLC using Aminex HPX-42A Carbohydrate column (Bio-Rad; 7.8 mm I.D. × 300 mm; 75 °C) with water at 0.5 ml/min, and a refractive index detector, RI-2031 Plus (Jasco).

From the reaction products, the trisaccharide and tetrasaccharide products were prepared. A reaction (10 ml) was made as above but with 174 nM MnLam55A for 24 h. The trisaccharide and tetrasaccharide were separated by gel-filtration column chromatography using Toyopearl HW-40s (Tosoh; 5 cm I.D. × 100 cm) with water under 1.8 ml/min and analyzed by ESI-MS using Exactive Plus (Thermo Fisher Scientific) and NMR as described above.

### Reaction with laminarioligoasccharides

A reaction mixture (50 μl) of 10 mM laminarioligosaccharides (DP2–6; Megazyme), 10 mM sodium acetate buffer (pH 5.6), 0.2 mg/ml BSA, and 17.4 nM MnLam55A was incubated at 30 °C for 180 min. Aliquots (10 μl) taken at several timepoints were heated at 100 °C for 3 min. The samples were analyzed by TLC as described above. Progress of initial reactions was monitored by analyzing samples (3–12 min, every 3 min) with high-performance anion-exchange chromatography with pulsed amperometric detection.

MS analysis of laminaripentaose-hydrolyzed products in H_2_O and H_2_^18^O was performed as follows. The reaction was made in a mixture (25 μl) of 2 mM laminaripentaose, 40 mM sodium acetate buffer (pH 5.6), 0.2 mg/ml BSA, and 139 nM MnLam55A in H_2_O or 99.2% (v/v) H_2_^18^O (Sigma) at 30 °C for 30 min. The samples were heated at 100 °C for 3 min, dried, and analyzed by ESI-MS as described above.

### Substrate specificity of MnLam55A

Reaction velocities with β-glucan were measured with the following substrates: laminarin, scleroglucan (Biosynth), curdlan (Fujifilm Wako Pure Chemical), paramylon (Sigma), and barley β1-3/1-4-glucan (Sigma). The reactions were made in a mixture (100 μl) of 1 mg/ml β-glucan, 40 mM sodium acetate buffer (pH 5.6), 0.2 mg/ml BSA, and MnLam55A (1.86 nM for laminarin and 186 nM for the others) at 30 °C for 15 min. The reaction for the kinetic analysis with laminarin was done in a reaction mixture (100 μl) of 0.2 to 2 mg/ml (6.4–64 μM) laminarin, 40 mM sodium acetate buffer (pH 5.6), 0.2 mg/ml BSA, and 3.48 nM MnLam55A, at 30 °C for 12 min. Reducing sugar was measured as described above. For laminarioligosaccharides (DP3–6), reactions were similar to those for laminarin but containing 2 to 10 mM laminarioligosaccharides and 34.8 nM MnLam55A, at 30 °C for 10 min. Release of d-glucose was quantified with a Glucose CⅡ Test (Fujifilm Wako Pure Chemical) (25, 26) after aliquots (25 μl), heated at 100 °C for 3 min, were mixed with 50 μl of 2 M Tris–HCl buffer (pH 7). Nonlinear regressions with the Michaelis–Menten equation on [S]-*v* plots were performed using Grafit version 7 (Erithacus Software, http://www.erithacus.com/grafit/). Three independent replications of each experiment were conducted.

### Crystallization and data collection

Crystallization of MnLam55A was prepared by the sitting drop vapor-diffusion method as follows: the drop (1.5 μl) was prepared by mixing 5.1 mg/ml MnLam55A in 10 mM Hepes-NaOH buffer (pH 7), and 10 mM glucose with the same volume of reservoir solution consisting of 0.1 M Hepes-NaOH buffer (pH 7), 1 M disodium succinate, and 10 mg/ml polyethylene glycol monomethyl ether 2000. Crystallization was observed within 37 days at 20 °C. Crystals were removed from the crystallization solution and flash-cooled. Diffraction data were collected on a beamline BL45XU at SPring-8. The datasets were indexed, integrated, scaled, and merged using the XDS program suite (https://xds.mr.mpg.de/) (27). The asymmetric unit of MnLam55A contained two molecules. Estimated Matthews coefficient and solvent content (28) were 3.09 Å^3^Da^−1^ and 60.2%, respectively. The data collection and processing statistics are summarized in Table S1.

### Structure solution and refinement

The structure of MnLam55A was determined by the molecular replacement method with the program AutoMR in the PHENIX program package (https://phenix-online.org/) (29, 30). The model structure constructed by ColabFold (31) was used as the search model. The refinement process was carried out using the program phenix.refine in conjunction with interactive fitting and rebuilding based on 2*F*_o_ − *F*_c_ and *F*_o_ − *F*_c_ electron densities using COOT (https://www.ccp4.ac.uk/) (29, 32). Water molecules were constructed based on electron densities. The crystal was pseudomerohedrally twinned with the twin operator (−h, −l, −k). The final refinement statistics and geometry defined by MOLPROBITY (33) are shown in Table S1. The atomic coordinates and structure factors were deposited in the Protein Data Bank (http://www.wwpdb.org/; PDB ID, 8JHH). All structure figures were generated by PyMOL ver. 2.6.0a0 (Schrödinger, LLC, https://pymol.org/).

### Docking simulation

A docking simulation was performed by AutoDock Vina (ADT version 1.5.6) (34) to estimate the binding mode of MnLam55A to the laminarin main chain. The structure of 6^Ⅲ^-*O*-glucosyl laminaritriose was constructed by BIOVIA Discovery Studio Visualizer (Dassault Systèmes BIOVIA). In the simulation, water and glycerol molecules in the structure of MnLam55 A were removed. The 6^Ⅲ^-*O*-glucosyl laminaritriose was docked into the active site of MnLam55A using the grid box with a spacing of 1 Å and dimension of 40 × 40 × 114, centered at positions of 0 (x), 0 (y), and 0 (z).

## Data availability

The data underlying this article are available upon reasonable request to the corresponding author.

## Supporting information

This article contains supporting information.

## Conflict of interest

The authors declare that they have no conflicts of interest with the contents of this article.
