# Supplementary material for: Molecular mechanism for endo-type action of glycoside hydrolase family 55 endo-β-1,3-glucanase on β1-3/1-6-glucan
Source: J Biol Chem. 2023 Sep 27;299(11):105294. doi: 10.1016/j.jbc.2023.105294 (PMC10637969; doi:10.1016/j.jbc.2023.105294)
Supplement: Supporting Figures S1 and S2 and Table S1 [file mmc1.docx]

Supporting information

Molecular mechanism for endo-type action of glycoside hydrolase family 55 endo-β-1,3-glucanase on β1-3/1-6-glucan

Tomoya Ota^1^, Wataru Saburi^1^, Takayoshi Tagami^1^, Jian Yu^2^, Shiro Komba^3^, Linda Elizabeth Jewell^4^, Tom Hsiang^5^, Ryozo Imai^6^, Min Yao^2^, Haruhide Mori^1†^

Fig. S1 s-2

Fig. S2 s-3

Table S1 s-4

**A**

**B**

**Fig. S1. Effect of pH and temperature on the activity of MnLam55A.**

(A) Effect of pH on recombinant MnLam55A activity. Open and closed circles show activity and stability, respectively. (B) Effect of temperature on recombinant MnLam55A activity. Open and closed circles show activity and stability, respectively.


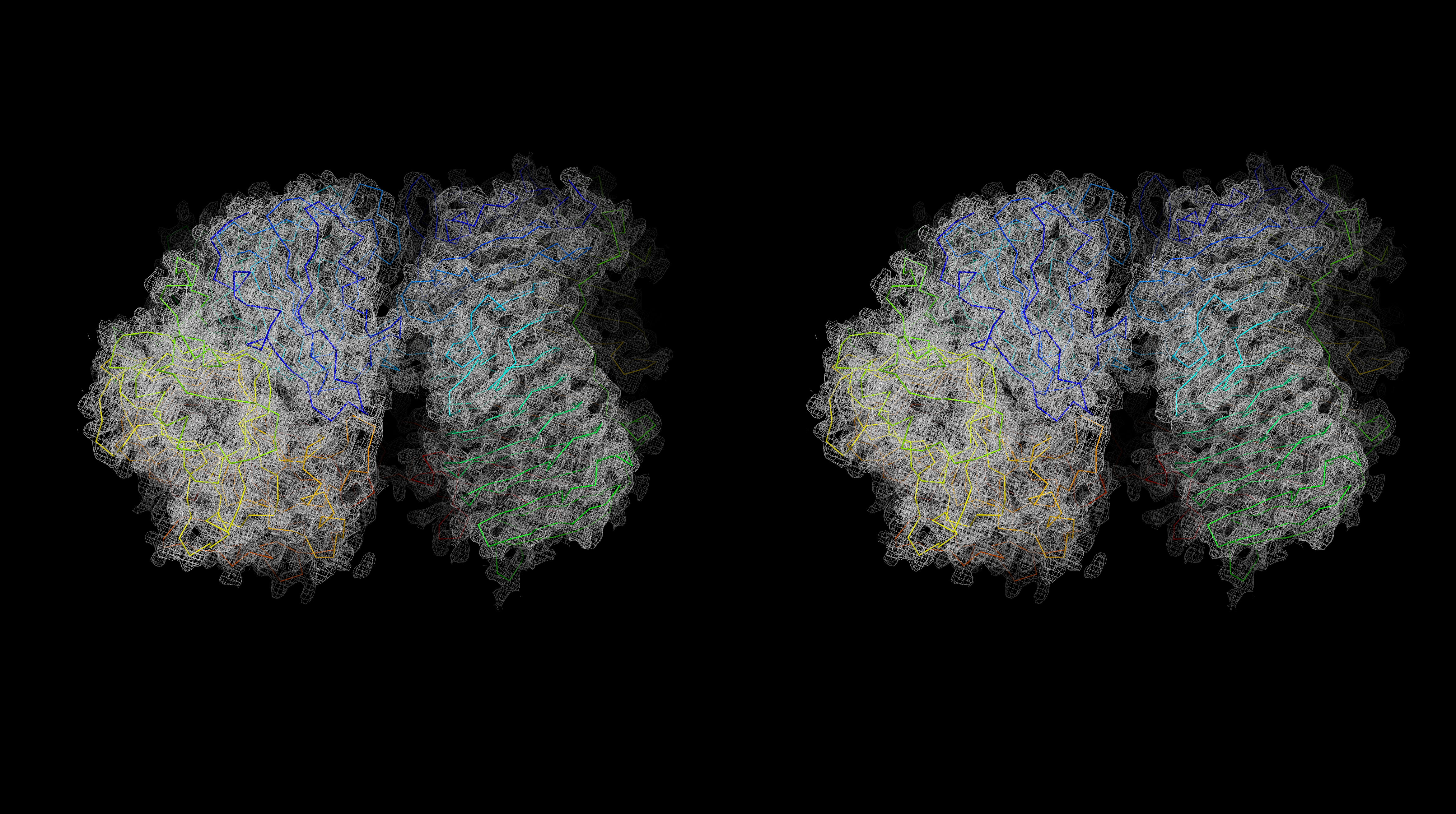


x-axis

180°


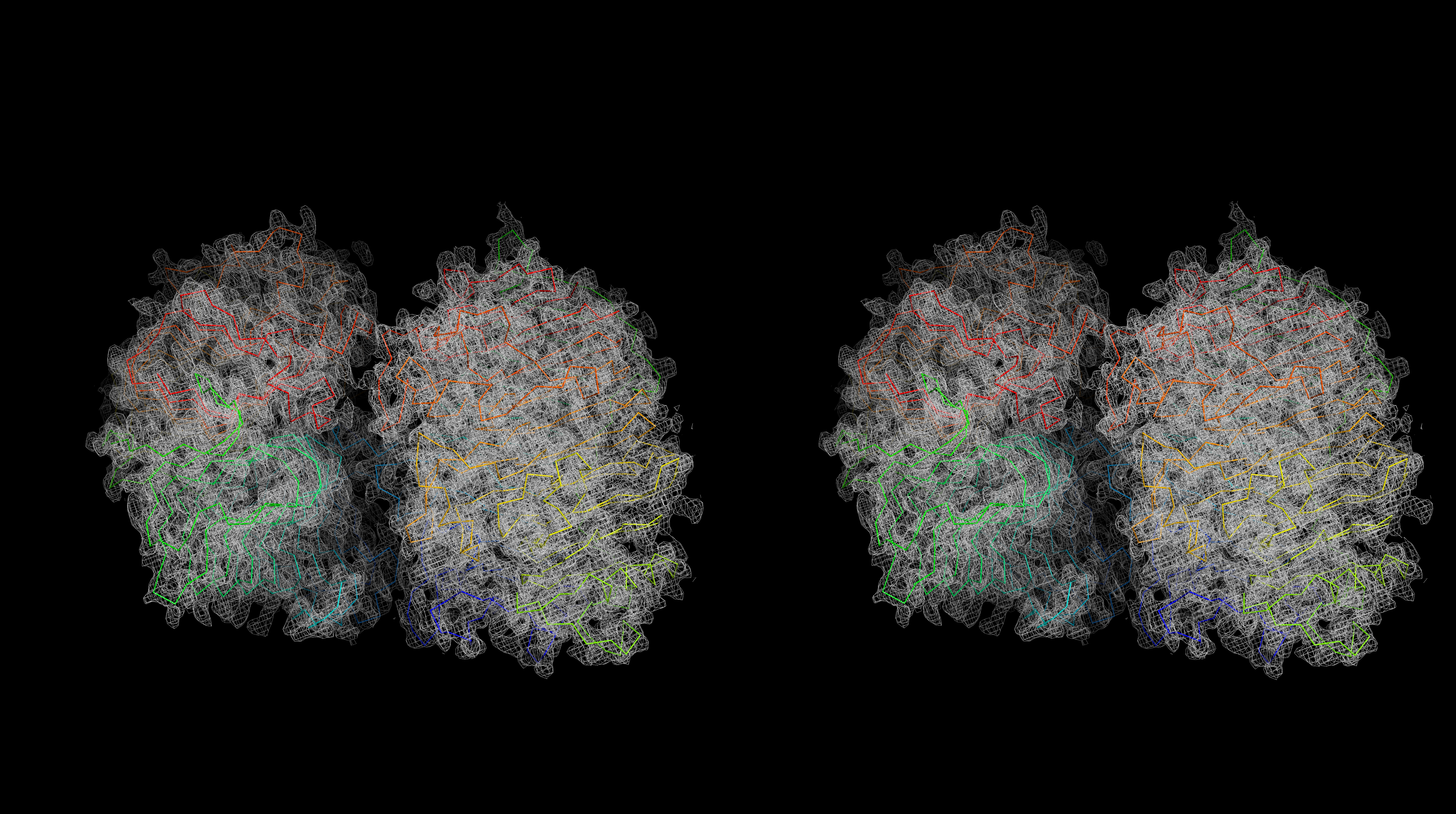


**Fig. S2. Electron density map of the crystal structure of MnLam55A.**

2Fo-Fc map of the crystal structure of MnLam55A in an asymmetric unit is drawn as stereo view. The counter level is 1 σ. Each monomer is shown as the spectrum from N (blue) to C (red) terminus.

**Table S1. Summary of crystallization conditions, data collection, and refinement statistics.**

|  | MnLam55A |
| --- | --- |
| **Data collection** |  |
| PDB ID | 8JHH |
| Beamline | SPring-8 BL45XU |
| Space group | *I*2_1_2_1_2_1_ |
| Unit cell parameters a, b, c, (Å) | 89.2, 211.0, 211.2 |
| Unit cell parameters α, β, γ, (º) | 90, 90, 90 |
| Wavelength (Å) | 1.0000 |
| Resolution range (Å) | 50.0-2.40 (2.55-2.40) |
| Total No. of reflections | 467,996 (77,596) |
| No. of unique reflections | 77,596 (12,330) |
| R_meas_ (%) | 27.1 (98.9) |
| R_merge_ (%) | 24.8 (90.0) |
| R_pim_ (%) | 11.0 (43.1) |
| <I/σ(I)> | 6.48 (1.68) |
| Wilson B-factor (Å^2^) | 27.6 |
| CC_1/2_ | 98.8 (78.1) |
| Completeness (%) | 99.8 (99.2) |
| Redundancy | 6.03 (5.87) |
|  |  |
| **Refinement** |  |
| No. reflections used in refinement | 77,596 |
| No. reflection used for R_free_ | 3,879 |
| R_work_/R_free_ (%) | 16.3/19.4 |
| Twin operator | −*h*, −*k*, −*l* |
| Twin fraction (%) | 36.8 |
| CC* | 99.5 (79.4) |
| CC_work_ | 88.6 (60.0) |
| CC_free_ | 85.3 (50.6) |
| No. of atoms |  |
| Macromolecules | 11,322 |
| Ligand/ion | 3 |
| Water | 236 |
| B-factors (Å^2^) |  |
| Macromolecules | 22.4 |
| Ligand/ion | 23.9 |
| Water | 31.2 |
| Protein residues | 1,470 |
| RMSD from ideal |  |
| Bond lengths (Å) | 0.004 |
| Bond angles (°) | 0.63 |
| Clashscore | 3.77 |
| Ramachandran |  |
| Favored (%) | 95.4 |
| Allowed (%) | 4.4 |
| Outliers (%) | 0.2 |

Values in parentheses are for the highest resolution shell.
